# Mutagenic and DNA repair activity in traffic policemen: a case-crossover study

**DOI:** 10.1186/s12995-018-0206-9

**Published:** 2018-08-08

**Authors:** Caterina Ledda, Carla Loreto, Massimo Bracci, Claudia Lombardo, Gaetano Romano, Diana Cinà, Nicola Mucci, Sergio Castorina, Venerando Rapisarda

**Affiliations:** 1 Occupational Medicine, Department of Clinical and Experimental Medicine, University of Catania, 95100 Catania, Italy; 2 Human Anatomy and Histology, Department of Biomedical and Biotechnology Sciences, University of Catania, 95100 Catania, Italy; 3 Occupational Medicine, Department of Clinical and Molecular Sciences, Polytechnic University of Marche, 60100 Ancona, Italy; 4 Clinical Pathology Unit, “Garibaldi Centro” Hospital of Catania, 95100 Catania, Italy; 5 Occupational Medicine, Department of Experimental and Clinical Medicine, University of Florence, 50100 Florence, Italy

**Keywords:** PAHs, DNA damage, Oxidative stress, Air pollution, Urban traffic, Cancer, Worker, Salmonella typhimurium, 8-oxodG, 1-hydroxypyrene

## Abstract

**Background:**

Emissions from vehicles are composed of heterogeneous mixtures of hazardous substances; several pollutants such as Polycyclic Aromatic Hydrocarbons (PAHs) are amongst the most dangerous substances detected in urban monitoring. A cohort of traffic policemen usually occupationally exposed to PAHs present in the urban environment were examined in order to assess the mutagenicity and DNA capacity repair.

**Methods:**

Seventy-two urban traffic policemen working in Catania’s metropolitan area were enrolled in the study. Two spot urine samples were collected from each subject during the whole working cycle as follows: sample 1 (S1), pre-shift on day 1; sample 2 (S2) post-shift on day 6. 1-hydroxypyrene (1-OHP) was measured to serve as an indirect exposure indicator. Urinary mutagenic activity was assessed through the plate incorporation pre-incubation technique with S9, using YG1024 Salmonella typhimurium strain over-sensitive to PAH metabolite. Concentrations of urinary 8-oxodG were measured using liquid chromatography tandem mass spectrometry.

**Results:**

As regards the exposure to PAHs, results highlighted a statistically significant difference (*p* < 0.001) between pre-shift on day 1 and post-shift on day 6 levels. Mutagenic activity was detected in 38 (66%) workers on S1 and in 47 (81%) on S2. Also 8-oxodG analysis showed a statistically significant difference between S1 and S2 sampling.

**Conclusions:**

This study demonstrated that occupational exposure to pollutants from traffic emission, assessed via 1-OHP measurements in urine, may lead to DNA repair and mutagenic activity, in line with other studies.

## Background

Urban traffic may impact on human health through various biological mechanisms and causes several health effects [1–5]. Moreover, the association between exposure to traffic-related air pollution and cause-specific mortality and morbidity has long been studied and dealt with in several epidemiological surveys [4, 6–9]. Emissions from vehicles are composed of heterogeneous mixtures of hazardous substances [10]; several pollutants such as Polycyclic Aromatic Hydrocarbons (PAHs) are amongst the most dangerous substances detected in urban monitoring [11–13].

PAHs are a large group of chemicals with 2 to 7 fused aromatic rings [14]. Benzo(a)pyrene (B(a)P) is one of the best-known PAHs, categorized by the International Agency for Research on Cancer (IARC) as carcinogenic to humans (group 1).

Besides, B(a)P is commonly used as indicator of PAHs global concentrations in environmental monitorings [15]. Many other PAHs are well known as cytotoxic, carcinogens, mutagens and teratogens and therefore represent a serious threat for the general population’s health and well-being [5, 14, 16]. Mutagenicity of PAHs, associated with urban traffic, has been demonstrated only through in-vitro assays [17–22]. Epidemiological studies of the correlation between presence of urinary mutagens and exposure to PAHs have been previously conducted [23–26] and certainly contributed to adapting the law limits on air pollution. Presently in Europe, Directive 2004/107/EC provides the target values for PAHs and establishes that the threshold limit is 1 ng/m^3^.

PAHs and other genotoxic chemicals are metabolized by humans and induce the expression of cytochrome P450 enzymes (i.e. CYP1A2) [27–29]. The CYP1A2 enzyme is involved in the metabolic activation of a wide range of chemicals and carcinogens like PAHs [28, 29]. Its activity has been shown to increase by smoking, ingestion of charbroiled meat, cruciferous vegetables, PAHs and PCBs exposures [28, 30–33]. The catalyzed metabolism by CYP1A2 can generate reactive oxygen species (ROS) which might lead to oxidative DNA damage [27, 34, 35]. This damage has been associated with an increased risk of cancer, generally ascribed to DNA adducts [36–38]. Oxidative DNA damage may be also important in carcinogenesis since the DNA base lesions, such as 8-oxo-7,8-dihydro-2′-deoxyguanosine (8-oxodG), are massive and highly mutagenic [39, 40]. However, DNA repair via nucleotide and base excision processes leads to elimination and excretion of 8-oxodG in urine quantitatively without metabolism [41–44]. Urinary excretion of 8-oxodG is the most widely used non-invasive urinary biomarker of oxidative stress and its measurement in urine has been proposed to assess whole-body DNA repair activity [43, 44].

In the present study, the authors investigated a cohort of traffic policemen usually occupationally exposed to PAHs present in the urban environment in order to assess the mutagenic and DNA repair activity.

## Methods

### Study designs, population and setting

In this case-crossover study the population comprised 72 urban traffic policemen working in Catania’s metropolitan area and spend > 6 h outdoor, daily. A working cycle consisted of six consecutive working days followed by two days off. All subjects enrolled in the study were of Caucasian origin, living in the same work area. Subjects had been current non-smokers for at least 6 months, their age ranging between 20 and 60 years, being employed during at least the same period, had no history of chronic or recent illnesses (diabetes or influenza for example) and had not been taking any medication (omeoprazole, for instance) that could interfere with the study results. The study was conducted in a framework of regular occupational medical visits in April – July 2016.

Several haematological parameters tested in all these policemen, including haemoglobin, haematocrit, platelets, white blood-cell count, lymphocytes and neutrophils were analysed following standard methods.

Two spot urine samples were collected from each subject during the whole working cycle as follows: sample 1 (S1), pre-shift on day 1; sample 2 (S2) post-shift on day 6.

Prior to this, a diet had been prescribed, two weeks before, so that it did not affect this study results over the whole sampling period. In particular, subjects were asked to avoid charcoal-cooked or grilled foods.

Urine samples from workers were collected in polyethylene containers and stored in the dark at − 20 °C until analysis.

### Creatinine analysis

Urine creatinine concentration was measured by spectrophotometry according to Jaffé (1885), using a commercial laboratory kit (Roche Diagnostics, Basel, Switzerland) [45].

### 1-hydroxypyrene analysis

The well-validated PAHs exposure biomarker 1-hydroxypyrene (1-OHP) was measured to serve as an indirect exposure indicator [46, 47]. Urinary 1-OHP was determined by HPLC (Agilent Technologies, Santa Clara, California, USA) with the fluorescence detection method, using a commercial laboratory kit (Chromsystems Instruments & Chemicals GmbH, Gräfelfing, Germany).

Briefly, after the enzymatic hydrolysis of each sample, carried out mixing 1 ml urine with 50 μl internal standard and 200 μl of the prepared enzyme solution in a reaction vial, a solid phase extraction was performed, by adding 1 ml of hydrolyzed urine to a Sample Clean Up Column and drawing through by centrifugation (2 min at 700 x g) or suction. Effluents were discarded. Then the sample was centrifuged (1 min at 700 x g) with 1000 μl Wash Buffer through the Sample Clean Up Column. Effluents were discarded. Then 300 μl of Elution Buffer were centrifuged (2 min at 700 x g) through the Sample Clean Up Column into a light-protected collection vessel. Finally, 10 μl eluate were injected into the HPLC system in isocratic flow rate (1.2 ml/min) at 35 °C. Wave lengths were 242 and 388, for excitation and emission, respectively. The 1-OHP levels were adjusted by urinary creatinine excretion and expressed as μg/g creatinine. The method limit of quantification was 0.1 μg/l.

### Mutagenic activity analysis

Urinary mutagenic activity was evaluated with the plate incorporation pre-incubation technique with S9, using YG1024 Salmonella typhimurium strain over-sensitive to PAH metabolite.

Urine samples (~ 50 ml) were thawed and filtered to remove urothelial cells. The exact volume of each sample was recorded and the samples enzymatically deconjugated in 0.2 M (10% v/v) sodium acetate buffer (pH 5.0) (Sigma–Aldrich, Missouri, USA), containing Helix pomatia β-glucuronidase (Sigma–Aldrich, Missouri, USA) 6 units/ml urine and 2 units/ml urine of sulphatase (Sigma–Aldrich, Missouri, USA) for 16 h at 37 °C. Solids were removed by centrifugation. Deconjugated urinary metabolites were then concentrated using solid-phase extraction on Mega Bond Elut Flash cartridge, C18, 2 g, 12 mL (Agilent Technologies, California, USA) with methanol elution (Sigma–Aldrich, Missouri, USA). The resulting extracts were reconstituted in dimethylsulphoxide (DMSO; Sigma–Aldrich, Missouri, USA) to produce urine extracts suitable for assessing mutagenicity. Extracts were stored at − 20 °C until use.

Urines were tested for mutagenic activity in the Salmonella assay mainly according to Maron and Ames [48].

Mutagenic activity was determined using the plate-incorporation pre-incubation technique on the YG1024 Salmonella typhimurium strain in the presence of Aroclor-induced rat liver S9 (50 μl per plate) [49]. The concentrations selected for mutagenicity testing were 0.3, 0.6, 1.2, 3.0 and 6.0 ml-equivalent urine per plate. 2-Aminofluorene (0.2 μg per plate) was used as a positive control and negative solvent checks (i.e. DMSO) were made on each day of mutagenicity testing.

Following a 72-h incubation at 37 °C, the frequency of mutant (i.e. revertant; rev) colonies was scored using a ProtoCol automated colony counter (Synbiosis Corporation, Exton, PA, USA).

Mutagenic activity was taken as positive when at least one of the tested doses was able to double the number of revertants compared to spontaneous ones and expressed as the slope of the linear portion of the dose–response curve calculated by the linear regression method, from at least two urine extract doses different from zero, as number of revertants/ml urine and number of revertants/mmol of creatinine.

### Oxidative DNA lesions analysis

Concentrations of urinary 8-oxodG were measured using liquid chromatography tandem mass spectrometry (LC–MS/MS). Briefly, 5 pmol of internal standard, 15 N5–8-oxodG, were added to 200 μL of urine and then ultrapure water was added to a final volume of 1 mL, prior to solid-phase extraction (Oasis HLB column, 1 mL, 30 mg; Waters, Connecticut, USA). Quantitative analysis was performed using LC–MS/MS (6420, Agilent Technologies, California, USA).

The urine samples were processed and analyzed in duplicates on two occasions and the repeatability of the method expressed as the coefficient of variation was 10.5%. The 8-oxodG concentration was calculated as the mean of the two measurements. Two internal controls were included in each batch. Urinary 8-oxodG concentrations were adjusted to the average-specific urine gravity (1.015 g/ml) using the formula: 8-oxodG x [(1.015–1)/(measured specific gravity − 1)], as well as to nmol 8-oxodG/mmol creatinine.

### Statistical analysis

Data were summarized as mean ± SD for continuous variables and frequencies for categorical variables. Normality was checked by Kolmogrov-Smirnov test and homogeneity of variance by Levene’s test. Unpaired *t* test was used to compare the means of two groups. *P* values ≤0.05 were considered significant. Correlations were evaluated using simple regression analysis. Data analysis was performed using GraphPad Prism ver.7 (GraphPad Software, Inc. USA).

## Results

Application of the exclusion criteria caused 14 workers to be ruled out of the sample because they admitted not having complied with the previously prescribed diet directives. Out of the 58 remaining subjects, 41 were males and 17 females, averagely aged 47 and with a mean working history of 17.6 years. Table 1 reports the main features of the subjects.

**Table 1:** Main features of samples and main results

	Results
Gender (Male)	41 (71%)
Age (yrs)	47 ± 10.2
BMI (kg/m^2^)	22.7 ± 2.1
Working age (yrs)	17.6 ± 8.4
No smokers	58 (100%)
Alcohol consumption (g/die)	12.4 ± 2.7

A routine occupational health examination revealed no alteration of any hematological parameters in any of the subjects. As regards the exposure to PAHs, results highlighted a statistically significant difference (*p* < 0.001) between pre-shift on day 1 and post-shift on day 6 in 1-OHP levels, that were 0.17 ± 0.09 and 0.38 ± 0.12 μg/g creatinine, respectively.

Mutagenic activity was detected in 38 (66%) workers on S1 and in 47 (81%) on S2, the mean levels of revertants/mmol creatinine being 21.26 ± 10.63 and 62.43 ± 20.91, respectively (*p* < 0.001).

Also 8-oxodG analysis showed a statistically significant difference (p < 0.001) between S1 and S2 sampling, the mean values being 2.73 ± 1.11 (nmol/mmol creatinine) and 4.52 ± 1.27 (nmol/mmol creatinine), respectively.

**Fig. 1:**
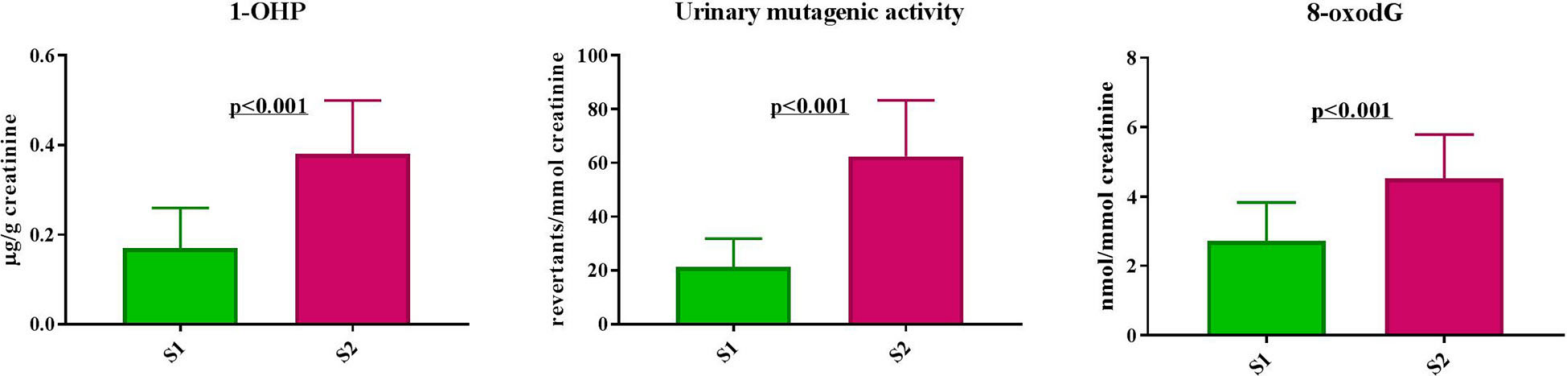
Plot of 1-OHP, mutagens and oxidative DNA lesions in traffic policemen

Figure 1 reports the graphic results.

No statistically different variation was detected between males and females for 1-OHP, mutagenic and repair activity.

Correlation between 1-OHP with mutagenic activity and 8-oxodG showed a statistically significant difference (*p* > 0.05).

## Discussion

Air pollution is a constant problem the world over and it has given rise to many different health problems [50]. As described by World Health Organization, traffic is the most important contributor to outdoor air pollution, because it is associated with negative effects on human health [51]. Traffic pollution contains high proportions of PAHs which is a group of over a hundred different organic compounds [10]. The main source of PAHs in the environment is incomplete combustion of organic substances; however, they are mainly released from vehicle exhaust pipes during diesel and petrol incomplete combustion [52, 53].

PAHs have drawn the scientific community’s attention because of their persistence and carcinogenic properties [54]. They enter the human body via lungs, ingestion and cutaneous paths of absorption [55]. In the urban environment, traffic pollution can be observed in traffic jams, while many studies provided evidence that owing to these events, traffic police workers, bus drivers and other cohorts exposed to traffic pollution are highly exposed to PAHs [56–58]. In human biomonitoring studies, the urinary 1-OHP, a metabolite of pyrene is a most widely used PAH internal dose biomarker and represents an internal dose of PAHs [59]. 1-OHP is also a biomarker of exposure to mixtures of PAHs [60]. A good correlation between PAH concentration in the air and urine 1-OHP has been observed in several occupational studies. Urinary levels of 1-OHP are often greatly increased in the population of polluted areas compared to those of less polluted ones [58, 60]. In addition to direct occupational exposure, indirect exposure to the outdoor environment is also a cause of increased urinary 1-OHP concentration [61].

In the present study, increased 1-OHP values were analyzed from S1 sample to S2 sample, that is after 6 working days following vehicle traffic in the streets. 1-OHP values turned out to be borderline compared to those provided by the law (0.3 μg/g creatinine).

1-OHP values were higher than those detected by Hansen et al. [58] in bus drivers (mean 0.19 μmol/mol creat) and mail carriers (mean 0.11 μmol/mol creat). The same authors, referring to a preliminary survey, had described an increased mutagenic activity in these categories of workers, although the numeric value was not comparable, as it was not reported. [58].

A study conducted by Hu et al. [57] on vehicle traffic environmental PHAs monitoring confirms that street policemen are highly exposed, even more than cooks, their levels of exposures being comparable to workers in the black carbon manufacturing industries and coke plants.

A study conducted by Burgaz et al. [56] on Ankara policemen revealed high concentrations of 1-OHP among non-smoking policemen. Comparing the data obtained with another control study, the authors concluded that occupational exposure to vehicle traffic PAHs is most evident.

Scientific past literature showed that PAHs present in traffic pollution may cause significant oxidative stress, which is characterized by an increased synthesis of free radicals [60]. Oxidative stress occurs during the imbalance between the syntheses of free radicals and antioxidants [62]. It seems that PAHs may be considered as major risk factors present in the automobile exhausts, which induce oxidative stress, as exposure to PAHs is associated with the increased production of free radicals [63]. Oxidative stress, on the other hand, may be one of the mechanisms behind many adverse health effects related to air pollution [64].

The health risks associated with exposure to PAHs is the consequence of a disturbance in the anti-oxidants defense system, resulting in significant oxidative stress [64], which is one of the major mechanisms behind the onset of cancer [65]. Large amounts of reactive oxygen species and many electrophiles are generated during the activation of PAHs by CYP450, which bind covalently with DNA and disturb cell homeostasis [66].

High antioxidants’ activity in exposed subjects shows that they suffer from oxidative stress, because PAHs are known for their potential to cause oxidative stress [67, 68].

As reported by Chao et al. [69], PAHs exert their biological effects probably through the generation of ROS. These excess ROSs can lead to oxidative DNA damage. Amongst the most abundant oxidatively damaged DNA is 8-oxodG, which was found to induce mutation through G to T transversion [70].

In this study, the 8-oxodG level in S2 sample, after 6 working days, was significantly greater than S1 (day 1).

In the past decade, several studies using 8-oxodG as an oxidative injury biomarker found significantly higher 8-oxodG levels in leukocyte DNA or urine of workers exposed to PAHs deriving from coke oven emissions compared to those of non-exposed workers [71, 72]. A previous study showed a significant correlation between urinary 1-OHP and 8-oxodG in coke oven workers [73].

In the present study 8-oxodG well correlated with urinary 1-OHP concentrations.

The urinary 8-oxodG levels were also highly correlated with urinary 1-OHP and not confounded by other variables. Zang et al. [74] measured leukocyte 8-oxodG levels in workers and found that high-exposed workers had even lower levels of 8-oxodG than low-exposed workers.

Urine sampling at the end of the working week was widely used for the policemen because urinary 1-OHP excretion levels were found to rise during a working week and its half-life ranges from 6 to 35 h [75].

Besides, the urinary 8-oxodG at the end of the working week (S2) showed relatively higher levels compared to day 1 (S1), as the biomarker of effect (e.g., 8-oxodG) not only reflects the exposure during a week-shift but also a much longer period of exposure [76].

There are some limitations to the present study. Firstly, the number of workers investigated was small because strict inclusion criteria to select the participants were applied. A second limitation was that there was no environmental sampling. Thirdly, no benzene determination was carried out either in the environment or amongst the biological exposure indicators. The benzene action shall be analyzed in a further study. Finally, biological monitoring of the study should also take other seasons than spring into due consideration [77–82]. As a matter of fact, a previous study, conducted in Catania, revealed greater concentrations of PAHs during winter time.

## Conclusions

From the result analysis of this study, it can be observed that 1-OHP urine concentration increases significantly in street policemen, after one week work shift. This reveals a certain exposure to PAHs. In the same way, the mutagenic activity initially observed (S1) in 66% of the sample turns out to be increased, as it involves over 80% of it.

The DNA repair activity, computed by measuring 8-oxidG concentrations, significantly increased between the S1 and S2 periods. Besides, 8-oxidg values significantly correlated with 1-OHP concentrations.

The analysis of other risk factors such as benzene, also emitted into the atmosphere through exhaust pipes, will further contribute to better understanding such phenomenon.

In conclusion, this study demonstrated that occupational exposure to pollutants from traffic emission is correlated to DNA repair and mutagenic activity.

## Abbreviations

1-OHP: 1-hydroxypyrene
8-oxodG: 8-oxo-7,8-dihydro-2′-deoxyguanosine
B(a)P: Benzo(a)pyrene
CAT: catalase
GSH: Gluthatione
GSHPx: glutatione-peroxidase
IARC: International Agency for Research on Cancer
PAHs: Polycyclic Aromatic Hydrocarbons
